# UL52 Primase Interactions in the Herpes Simplex Virus 1 Helicase-Primase Are Affected by Antiviral Compounds and Mutations Causing Drug Resistance*

**DOI:** 10.1074/jbc.M114.609453

**Published:** 2014-10-02

**Authors:** Isabella Muylaert, Zhiyuan Zhao, Per Elias

**Affiliations:** From the Institute of Biomedicine, Department of Medical Biochemistry and Cell Biology, Sahlgrenska Academy, University of Gothenburg, Box 440, SE-405 30 Gothenburg, Sweden

**Keywords:** Antiviral Agent, DNA Primase, DNA Replication, Herpesvirus, Viral Replication

## Abstract

Herpes simplex virus 1 (HSV-1) UL5/8/52 helicase-primase complex is required for DNA unwinding at the replication fork and synthesis of primers during virus replication, and it has become a promising novel target for antiviral therapy. Using molecular cloning, we have identified three separate domains of UL52. Co-immunoprecipitation experiments in extracts from cells transiently expressing HA-tagged UL5, FLAG-UL8, and enhanced GFP-tagged UL52 domains revealed that the N-terminal domain of UL52 primase binds UL5 helicase and the middle domain interacts with the UL8 accessory protein. In addition, an interaction between the single strand DNA-binding protein ICP8 and the UL52 middle domain was observed. The complex between UL5 and UL52 was stabilized by the antiviral compound BAY 54-6322, and mutations providing resistance to the drug obliterate this effect. Our results also suggest a mechanism for accommodating conformational strain resulting from movement of UL5 and UL52 in opposite directions on the lagging strand template, and they identify molecular complexes that can be further examined by structural biology techniques to resolve the mechanism of primer synthesis during herpesvirus replication. Finally, they help to explain the mechanism of action of a novel class of antiviral compounds currently being evaluated in clinical trials.

## Teaser

**Background:** Herpes simplex virus DNA synthesis requires seven interacting virus proteins.

**Results:** Mapping revealed an N-terminal domain of UL52 primase interacting with UL5 helicase; a UL52 middle domain binds UL8 and the UL29 single strand DNA-binding protein.

**Conclusion:** Protein-protein interactions in the helicase-primase were affected by an antiviral compound and resistance mutations.

**Significance:** This work provides understanding of antiviral inhibition of replisome function.

## Introduction

Herpes simplex virus type 1 (HSV-1) is one of eight different herpesviruses infecting humans. An initial infection is accompanied by establishment of latency in neuronal cells from which the virus can be reactivated. The ability to establish latent infections is shared by all herpesviruses, and as a consequence, the prevalence of herpesvirus infections is very high in the adult population. Primary infections by HSV-1 and other herpesviruses can cause serious disease, and reactivation of latent virus may occur during treatments requiring efficient immune suppression. HSV-1 infections in immunocompetent individuals can be efficiently controlled by acyclovir, a deoxyguanosine analog, which after phosphorylation by a viral thymidine kinase acts as an efficient chain terminator capturing the viral DNA polymerase in a salt-stable complex (1–4). Immunocompromised individuals, however, experience a substantial risk for development of drug resistance (5, 6). Viral DNA replication is also a prime target for new generations of antiviral compounds. Several new drug candidates have recently been developed targeting the helicase-primase component of the HSV-1 replication machinery (7), and one of these, pritelivir or BAY 57-1293, has been shown to reduce HSV-2 virus production and cause a reduction of genital lesions in a clinical study (8). However, knowledge about the mechanism of action of the new generation of antiherpetic drugs and how mutations may cause drug resistance is still limited.

Replication of HSV-1 DNA depends on seven proteins encoded by the viral genome (9, 10). The origin binding protein encoded by the *ul9* gene binds and unwinds double-stranded DNA at the origins of replication together with the single strand DNA-binding protein ICP8 or UL29 protein (11). The remaining viral replisome components UL30-UL42 DNA polymerase and the trimeric helicase-primase complex are then assumed to be recruited to the activated origins of replication. The replisome can be operationally defined as a molecular machine inasmuch as none of its components can be exchanged for a functional homolog from a closely related virus (12). HSV-1 helicase-primase was first discovered as a DNA-dependent ATPase and subsequently shown to consist of the UL5 helicase, the UL8 accessory protein, and the UL52 primase subunits (13, 14). The helicase-primase complex appears to have its evolutionary origin in predecessors of Pif 1 helicase and in the archaeo-eukaryotic primase superfamily (15, 16). Intriguingly, UL8 is related to an inactivated family B DNA polymerase (17). A subcomplex consisting of UL5 and UL52 complexes retains helicase and primase activities but cannot support leading and lagging strand synthesis in the presence of the single strand DNA-binding protein ICP8 (18–21). The UL52-UL8 subcomplex, which cannot synthesize primers on single-stranded DNA, can still, albeit inefficiently, elongate RNA primers (22). At the replication fork, the UL5 helicase moves along lagging strand template DNA in a 5′–3′ direction. The UL52 primase, however, will lay down primers in the opposite direction. To perform these tasks, helicase-primase will most likely undergo a series of conformational changes triggered by interactions with the DNA template and/or by protein interactions between the three subunits and with the other enzymes involved in DNA synthesis. Little is known about these interactions, but it has been shown in yeast two-hybrid experiments that the UL8 protein interacts with the C-terminal 2/3 of the UL52 primase and that a conserved sequence motif in the C-terminal part of UL8 is required for the UL8-UL52 interaction (12, 23). Other protein-protein interactions within the helicase-primase complex as well as between helicase-primase and remaining replisome components remain to be elucidated.

Here, we performed experiments to map domains of the UL52 primase and their interactions with the UL5 helicase and the UL8 protein. In addition, we analyzed the effects of UL5 and UL52 resistance mutations to the HSV-1 helicase-primase inhibitors ASP2151 and BAY 54-6322 on the complex formation. We found that UL52 can be divided into three parts referred to here as the N-terminal (N), middle (M), and C-terminal (C) domains. The UL8 protein interacts exclusively with the M domain of UL52, whereas the UL5 protein interacts strongly with the N-domain and possibly with the C-domain. UL5 also seems to enhance the interaction between full-length UL52 and UL8. Resistance mutations within the N-domain severely impaired complex formation between UL5 and UL52. On the other hand, the antiviral compound BAY 54-6322 greatly enhanced the UL5-UL52 interaction, but this effect could be efficiently counteracted by the resistance mutations in the UL5 protein and the C-domain of UL52. Finally, we note that co-expression of the UL52 M-domain, UL8, and ICP8 leads to co-localization of these proteins into prominent intranuclear foci suggesting direct interactions between ICP8, UL8, and UL52. These studies provide the first detailed interaction map for the helicase-primase complex, and they also suggest a number of dynamic structural changes within the complex with mechanistic implications for drug action and development.

## EXPERIMENTAL PROCEDURES

### 

#### 

##### Cells

BHK^2^ cells were grown in Glasgow minimum essential medium supplemented with 10% fetal bovine serum (FBS, Invitrogen).

##### Plasmid Constructs

eGFP-tagged UL52 was made by fusion of the eGFP to the N terminus of HSV-1 UL52. A PCR-amplified megaprimer, consisting of the entire eGFP sequence flanked by short sequences overlapping the vector PE52 (24) at the insertion region, was generated and subsequently inserted into the vector by using the QuikChange II XL site-directed mutagenesis kit (Agilent Technologies). Similarly, HA-tagged UL5 was created by direct insertion of the HA-coding sequence right upstream the *ul5* gene in the PE5 vector (24). The FLAG- and emerald GFP (emGFP)-tagged UL8 constructs were as described previously (12). All HA-UL5 and eGFP-UL52 mutants were made using the kit referred to above, and all DNA oligonucleotides were produced by Eurofins Genomics.

##### DNA Synthesis Analysis

BHK monolayers in 24-well plates were transfected with the plasmids expressing the seven essential HSV-1 DNA replication enzymes together with the plasmid pUCOriS using Lipofectamine and Plus reagents (Invitrogen) as described previously (12). Cells were incubated at 37 °C, and after 48 h total DNA was isolated using the QIAamp DNA blood minikit (Qiagen). To measure total DNA and replicated pUCOriS DNA separately, each DNA was transferred in identical aliquots to two separate identical tubes. DNA was then either cleaved by the restriction enzyme HindIII alone or with HindIII and DpnI at 37 °C for 3 h. Total pUCOriS DNA (HindIII-cleaved) and newly synthesized DNA (HindIII- and DpnI-cleaved) were quantified by quantitative PCR, using iQ SYBR Green Supermix (Bio-Rad) with the forward primer, 5′-CAT TTT GCC TTC CTG TTT TTG CTC ACC-3′, and the reverse primer, 5′-GCT GT CAG ATC CAG TTC GAT GTA ACCC-3′. The DNA replication efficiency was calculated by taking the ratio between newly synthesized and total DNA. All measurements were done in three independent experiments.

##### Co-immunoprecipitation and Western Blot Assays

BHK monolayers were transfected with the indicated plasmids using Lipofectamine and Plus reagents (Invitrogen). At 20 h post-transfection, the cells were lysed, and the cleared extract was subjected to immunoprecipitation and subsequent Western blot analyses as described previously (12).

Expression levels were monitored by using 1/20th of the amount used for immunoprecipitation and labeled as “input” in the figures. When indicated, the helicase-primase inhibitor BAY 54-6322 (kindly provided by Alexander Birkmann, AiCuris) was added to the cell culture media immediately after the transfection mixture and to the cell lysate used for immunoprecipitation. EZView red anti-FLAG M2 (F7425, Sigma), EZView red anti-HA (E-6779, Sigma), and anti-GFP mAb-agarose (MBL) affinity beads were used for FLAG, HA, and GFP pulldown experiments, as described by the manufacturer. For ICP8 immunoprecipitation assays, a rabbit polyclonal antiserum to HSV-1 ICP8 was added to the cell lysate and incubated overnight at 4 °C followed by an additional 2 h of incubation with immobilized protein A/G resin (Thermo Scientific) and several washes, as recommended by the manufacturer. For detection of eGFP-tagged proteins, FLAG-UL8, HA-UL5, ICP8, and actin in Western blots, anti-GFP (ab290, Abcam), anti-FLAG (F7425 and F3165, Sigma), anti-HA (SAB4300603 and H9658, Sigma), and anti-HSV-1 ICP8 (ab20194, Abcam), a rabbit polyclonal antiserum to HSV-1 ICP8 and anti-actin (ab6276, Abcam) antibodies was used. Proteins were detected and quantified by chemiluminescence using Bio-Rad Clarity Western ECL substrate and Bio-Rad ChemiDoc XRS imager. Restore Plus Western blot Stripping Buffer (Thermo Scientific) was used for membrane stripping.

##### Immunofluorescence Analyses

BHK cells were seeded on ibiTreated 8-well μ-slides (Ibidi) and transfected with the indicated plasmids. After 16 h of incubation at 37 °C, the cells were fixed with cold methanol and incubated with either anti-HSV-1 ICP8 (ab20194, Abcam), anti-HA (H9658), or anti-FLAG (F3165) followed by incubation with Alexa Fluor 568-labeled secondary antibody (A11004, Invitrogen). Finally, the cell nucleus was stained with DAPI (Invitrogen). Confocal images were acquired using a Carl Zeiss LSM 700 Axio Observer. Z1 inverted the confocal laser-scanning microscope with either a plan-apochromat 20×/0.8 objective or with a plan-apochromat 63×/1.4 oil objective. Merging of images and profile plot measurements were done with the program ImageJ. Images were further processed for publication with Adobe Illustrator CS6.

## RESULTS

### 

#### 

##### Middle Domain of UL52 Binds UL8

**FIGURE 1.**
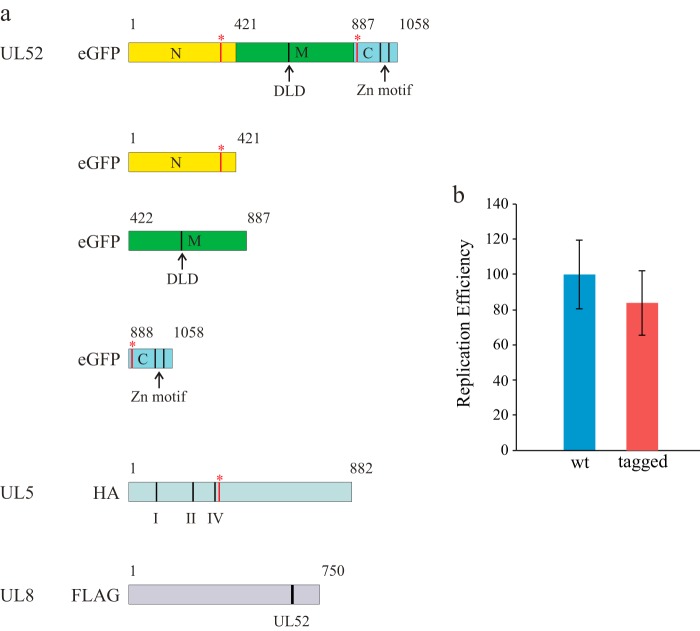
*a,* UL52, UL5, and UL8 components of the HSV-1 helicase-primase complex. For eGFP-tagged UL52, the N-, M-, and C-domains of HSV-1 UL52 defined by molecular cloning and interaction studies are shown in *yellow, green,* and *blue,* respectively. The numbers refer to amino acids in the primary sequence of the full-length proteins. The *red bars* with *asterisks* indicate mutations in UL52 causing resistance to helicase-primase inhibitors (S364, R367H, and A899T). The *black bar* in the M-domain indicates the position of the D*X*D motif found at the active site for primases. The *black bar* in the C-domain shows the positions of the C*XX*C and H*XX*C motifs indicative of zinc binding. Also shown are the GFP fusion proteins used for interaction studies. For HA-tagged UL5, the positions of helicase motifs I and II (Walker A and B boxes) together with motif IV as defined by Zhu and Weller (31) are shown as *black bars*. Resistance mutations (G352V, M355I, and K356T) are shown as a *red bar* with an *asterisk*. For FLAG-tagged UL8, the positions of mutations affecting DNA replication and interaction with UL52 primase are shown as a *black bar. b,* transient expression of plasmids encoding the untagged or tagged proteins eGFP-UL52, HA-UL5, and FLAG-UL8, together with UL9, UL29, UL30, and UL42 is able to support the replication of a plasmid containing the HSV-1 origin of replication. Triplicate experiments were performed. The difference between experiments using untagged proteins (*blue*) *versus* tagged proteins (*red*) was not statistically significant (Student's *t* test 0.41).

**FIGURE 2.**
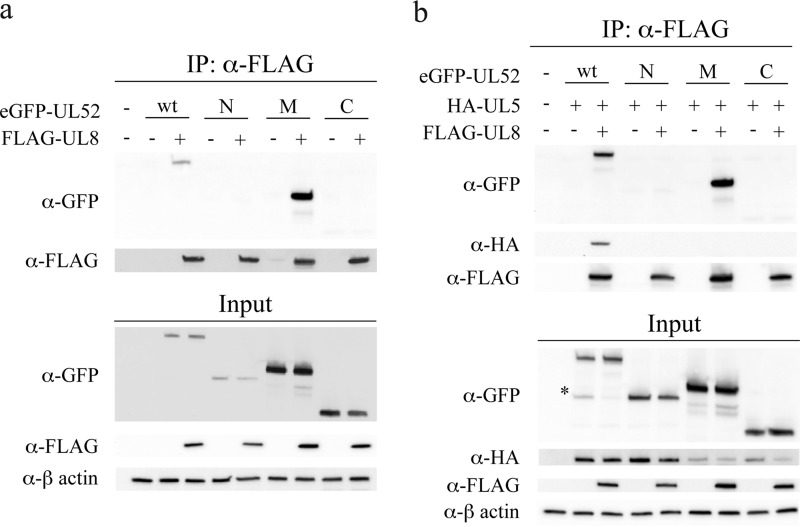
**UL8 binds to the UL52 M-domain.** *a,* Western blot analysis of an anti-FLAG co-immunoprecipitation (*IP*) experiment performed in cell extracts containing transiently expressed FLAG-UL8, eGFP-UL52, as well as eGFP-tagged N-, M-, and C-domains as indicated. *b,* Western blot analysis of an anti-FLAG co-immunoprecipitation experiment performed in cell extracts containing transiently expressed FLAG-UL8, HA-UL5, eGFP-UL52, as well as eGFP-tagged N-, M-, and C-domains as indicated. The *asterisk* shows the eGFP-UL52 fragment produced by endoproteolytic cleavage.

To identify molecular interactions within the helicase-primase complex, we divided the UL52 protein into distinct domains by molecular cloning (Fig. 1). Our starting point was an observation by Constantin and Dodson (23) demonstrating by yeast two-hybrid analysis an interaction between the last 2/3 of the UL52 primase (amino acids 350–914) and the UL8 protein. We then carefully defined the borders of the UL8-binding domain by performing small step truncations of the N and C ends. These constructs were transiently expressed in cells with an N-terminal eGFP tag and examined for the ability to co-immunoprecipitate with FLAG-UL8. A minimal UL8-binding domain, from here on referred to as the M-domain, consisting of amino acids 422–887 in UL52 could be identified with this experimental strategy (Fig. 1 and Table 1). The remaining N- and C-domains of UL52 were also eGFP-tagged and defined as consisting of amino acids 1–421 and 888–1058, respectively. We then examined the ability of these three domains to interact with FLAG-UL8 (Fig. 2). The M-domain was expressed well in cultured cells, and it was efficiently co-immunoprecipitated with FLAG-UL8 (Fig. 2*a*). In contrast, the N-domain was found in lower amounts in the extract, and neither the N-domain nor the C-domain was precipitated together with FLAG-UL8. We also looked at the influence of HA-UL5 on complex formation (Fig. 2*b*). We found that HA-UL5 was co-immunoprecipitated with FLAG-UL8 only when full-length eGFP-UL52 was present but not with the smaller eGFP-tagged M-domain. Interestingly, addition of HA-UL5 resulted in a reproducible 2-fold increase in the amount of full-length eGFP-UL52 in the precipitate suggesting that the presence of UL5 made it more accessible to FLAG-UL8. In this experiment, we also observed a shorter form of GFP-UL52, which probably is a result of proteolysis enhanced by the presence of HA-UL5 (Fig. 2*b*). This finding will be further discussed below.

**TABLE 1:** **eGFP-UL52 constructs and their interactions with UL8 and UL5** The final N-, M-, and C-domains are indicated in the table. Strong, medium, weak, and no interactions are indicated as +++, ++, +, and −, respectively, and inferred from the amount of co-immunoprecipitated protein in pulldown experiments.

eGFP-UL52 constructs	UL8 interaction		UL5 interaction
	−UL5	+UL5	
eGFP-UL52	++	+++	+++
Δ367–1058	−	−	+
Δ1–366; Δ915–1058	+++	+++	+
Δ1–914	-	-	+
Δ1–366	+++	+++	+
Δ367–914	−	−	−
Δ915–1058	++	+++	+++
Δ1–476; Δ915–1058	−	Not tested	Not tested
Δ1–461; Δ915–1058	−	Not tested	Not tested
Δ1–451; Δ915–1058	−	Not tested	Not tested
Δ1–441; Δ915–1058	−	Not tested	Not tested
Δ1–436; Δ915–1058	−	Not tested	Not tested
Δ1–431; Δ915–1058	−	Not tested	Not tested
Δ1–426; Δ915–1058	−	Not tested	Not tested
Δ1–421; Δ915–1058	+++	Not tested	Not tested
Δ1–421; Δ906–1058	+++	Not tested	Not tested
Δ1–421; Δ897–1058	+++	Not tested	Not tested
Δ1–421; Δ891–1058	+++	Not tested	Not tested
Δ1–421; Δ888–1058 (M-domain)	+++	+++	+
Δ1–421; Δ885–1058	−	Not tested	Not tested
Δ1–421; Δ879–1058	−	Not tested	Not tested
Δ1–421; Δ870–1058	−	Not tested	Not tested
Δ422–1058 (N-domain)	−	−	+++
Δ1–887 (C-domain)	−	−	+

**FIGURE 3.**
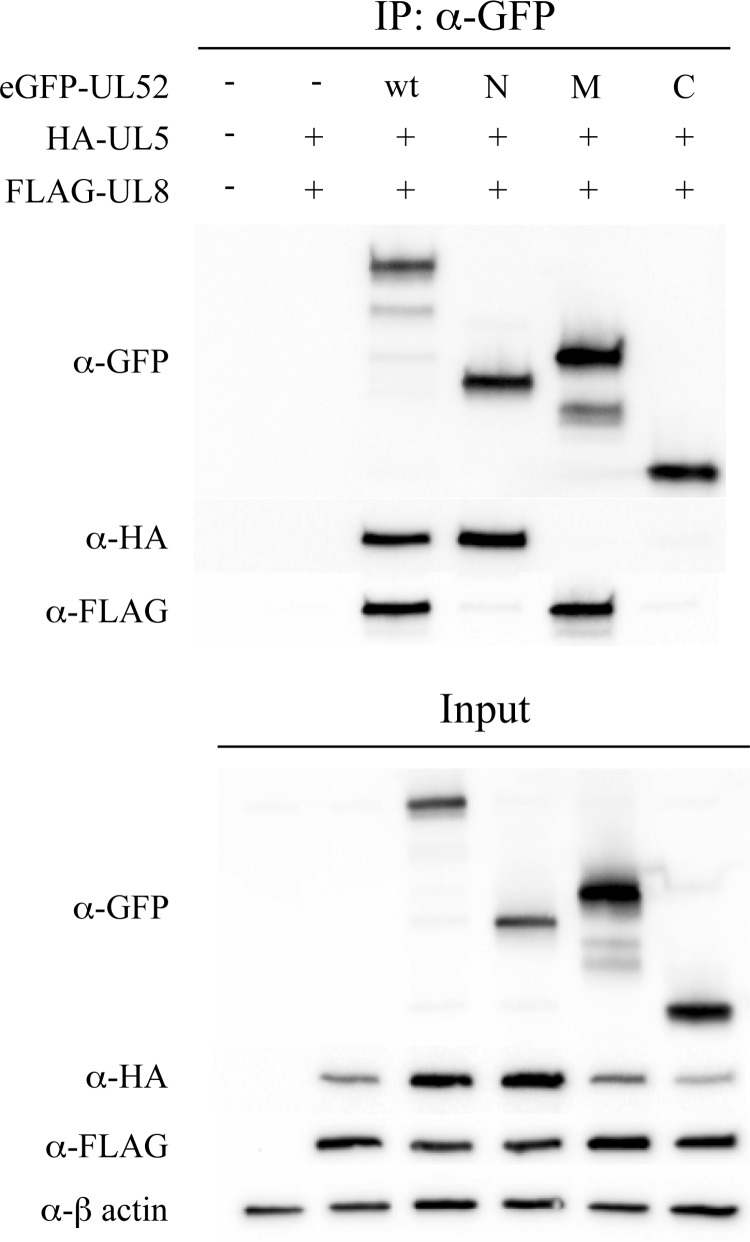
**Interactions between the eGFP-tagged N-, M-, and C-domains, UL5 and UL8 proteins.** Western blot analysis of an anti-GFP co-immunoprecipitation assay performed in cell extracts containing transiently expressed FLAG-UL8, HA-UL5, eGFP-tagged UL52 or eGFP-tagged N-, M- or C-domains, as indicated.

It is also worth noting that lower amounts of HA-UL5 were found in experiments using the transiently expressed M- and C-domains as compared with the N-domain and full-length UL52 (Fig. 2*b*). This observation probably reflects decreased solubility and/or stability of the protein in the absence of the appropriate interaction partner. Further truncation of the M-domain resulted in fragments unable to bind UL8, and such fragments were also expressed less efficiently suggesting that they were unstable (Table 1). Finally, in an anti-GFP co-immunoprecipitation assay, we found that the eGFP-tagged M-domain co-immunoprecipitated with FLAG-UL8 but not HA-UL5 (Fig. 3).

Together, these observations suggest that the M-domain is the primary interaction partner for UL8 and that access to the M-domain may be controlled by the N-domain and modified by the presence of UL5.

##### N-terminal Domain of UL52 Binds UL5

**FIGURE 4.**
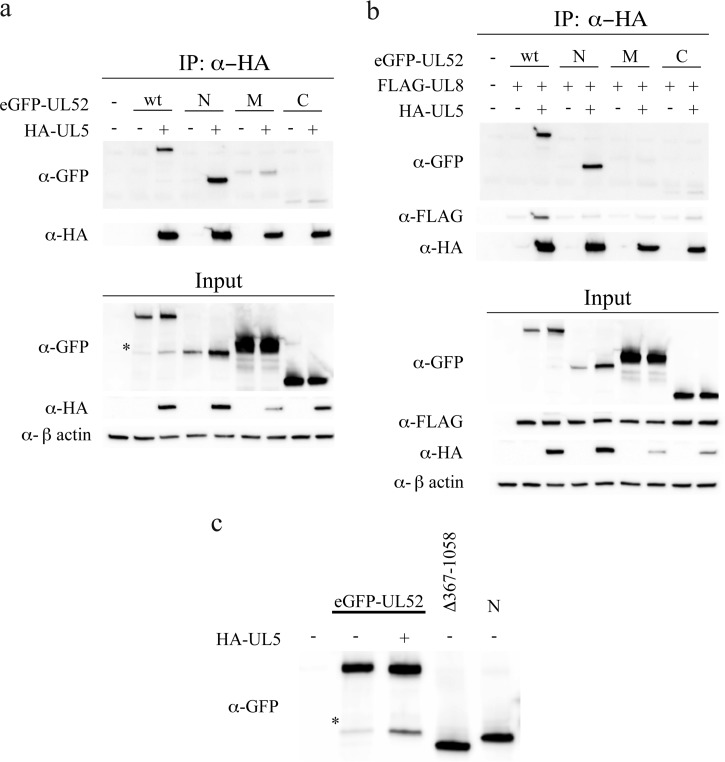
**UL5 binds to the N-domain of UL52.** *a,* Western blot analysis of an anti-HA co-immunoprecipitation (*IP*) experiment performed in cell extracts containing transiently expressed HA-UL5, eGFP-UL52, as well as eGFP-tagged N-, M-, and C-domains as indicated. An *asterisk* shows the eGFP-UL52 fragment produced by endoproteolytic cleavage in transfected cells. *b,* Western blot analysis of an anti-HA co-immunoprecipitation experiment performed in cell extracts containing transiently expressed HA-UL5, FLAG-UL8, as well as eGFP-tagged N-, M- and C-domains as indicated. *c,* mapping of a proteolytic fragment of eGFP-UL52. Anti-GFP Western blot of mock-transfected or transfected cells with expression plasmids encoding eGFP-tagged UL52 alone or together with UL5. eGFP-tagged Δ367–1058 and eGFP-tagged N-domain are used as size markers.

To investigate the interactions between UL5 and UL52, we made use of the N-, M-, and C-domains as defined above. Using HA-UL5 expressed together with eGFP-UL52 and eGFP-tagged N-, M-, and C-domains, we observed that HA-UL5 efficiently co-immunoprecipitated the full-length UL52 and even more efficiently the UL52 N-domain (Fig. 4*a*). Only very small amounts of the M- and C-domains were found in the immunoprecipitate. Therefore, we conclude that the N-domain is responsible for the formation of a stable UL5-UL52 complex. We also found lower amounts of HA-UL5 in the extract when HA-UL5 was expressed together with the M-domain indicating reduced stability in the absence of its interaction partner. Interestingly, higher amounts of HA-UL5 were found when co-expressed with the C-domain indicating, possibly, a stabilizing effect on UL5 (Fig. 4*a*).

The UL5-UL52 interaction could be further defined by the observation that the eGFP-tagged deletion mutant UL52 (Δ367–1058) did not bind HA-UL5 (Table 1). Thus, amino acids between position 367 and 421 appear to contribute significantly to the UL5-UL52 interaction.

We further investigated the effect of UL8 on UL5-UL52 complex formation in co-transfection experiments (Fig. 4*b*). We found again efficient co-immunoprecipitation of the N-domain and full-length UL52. Interestingly, the small amounts of M- and C-domains precipitated in the absence of UL8 were significantly reduced in its presence (Fig. 4, *a* and *b*). We also found that FLAG-UL8 was co-immunoprecipitated with HA-UL5 only when full-length eGFP-UL52 was present but not in the presence of the eGFP-tagged N-domain. Finally, HA-UL5 appeared to be unstable in the presence of only the M- and C-domains. As described in the preceding paragraph, we also performed a co-immunoprecipitation experiment using anti-GFP beads (Fig. 3). The results confirmed that only full-length eGFP-UL52 and the eGFP-tagged N-domain could bring down HA-UL5.

We noted again the appearance of a short fragment of eGFP-UL52 in these experiments (Fig. 4*a*). We made an attempt to map the putative cleavage site by expressing full-length eGFP-tagged UL52 in the presence or absence of HA-UL5. We used as size markers UL52 (Δ367–1058) and the eGFP-tagged N-domain and observed that the proteolytic fragment was, approximately, the same size as the eGFP-tagged N-domain (Fig. 4*c*). The result suggests the existence of a region in UL52 that is sensitive to endogenous proteases and is located at the border between the N- and M-domains. Exposure of this sequence appears to be enhanced in the presence of UL5 suggesting that it serves as a hinge region for conformational changes in UL52 induced by UL5.

To conclude, HA-UL5 interacts strongly with the N-domain of UL52 and in particular with amino acids 367–421. The C-domain, on the other hand, does not form a stable complex with UL5 but may enhance the solubility of that protein.

##### Interaction between UL5 and UL52 Is Affected by Mutations Found in Viruses Resistant to Helicase-Primase Inhibitors and Enhanced by the Drug BAY 54-6322

**FIGURE 5.**
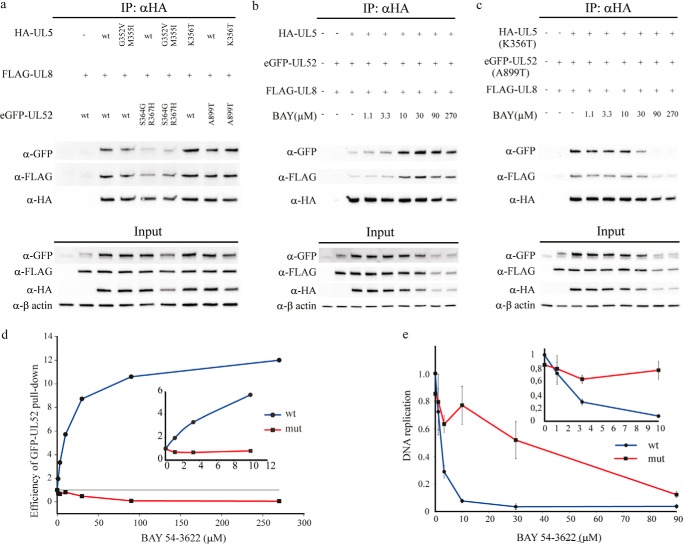
**UL5-UL52 interaction is affected by the BAY 54-6322 inhibitor as well as mutations causing drug resistance.** *a,* Western blot analysis of an anti-HA co-immunoprecipitation (*IP*) experiment performed in cell extracts containing transiently expressed FLAG-UL8, eGFP-UL52, and HA-UL5 as indicated. For HA-UL5, the wild type protein, a G352V/M355I double mutant, and a K356T single mutant were used. For eGFP-UL52, the wild type protein, an S364G/R367H double mutant, and an A899T single mutant were used. *b,* Western blot analysis of an anti-HA co-immunoprecipitation experiment performed in cell extracts containing transiently expressed FLAG-UL8, eGFP-UL52, and HA-UL5 as indicated. BAY 54-6322 was included as indicated. *c,* Western blot analysis of an anti-HA co-immunoprecipitation experiment performed in cell extracts containing transiently expressed FLAG-UL8, eGFP-UL52 (A899T), and HA-UL5 (K356T) as indicated. BAY 54-6322 was included as indicated. *d,* efficiency of eGFP-UL52 pulldown was measured from the experiments shown in *b* and *c* above by calculating ratios between eGFP-UL52 and HA-UL5 in the pulldown sample. The values were normalized to the ratio obtained in the absence of the drug. The inserted *graph* is magnifying the window between the concentrations 0 and 10 μm of the added drug. *e,* inhibition of oriS-dependent replication was measured in transfected cells using either WT or mutants HA-UL5 (K356T) and e-GFP-UL52 (A899T) together with WT expression plasmids for the remaining five HSV-1 replication proteins and pUCoriS as described under “Experimental Procedures.” The inserted *graph* is magnifying the window between the concentrations 0 and 10 μm of the added drug.

We noted that mutations in UL52 causing resistance to HSV-1 helicase-primase inhibitors lie within the N- and C-domains. More specifically, mutations in the N-domain (S364G and R367H) combined with mutations in UL5 (G352V and M355I) confer a 3000-fold resistance to ASP2151 (25), and the mutations A899T in the C-domain combined with K356T in UL5 result in a 2500-fold resistance to BAY 57-1293 (26). These observations suggest that the inhibitors are likely to affect the interactions between UL5 and UL52. To further analyze these effects, we performed anti-HA co-immunoprecipitation experiments using HA-UL5, FLAG-UL8, and eGFP-UL52 (Fig. 5).

We first looked at the effects of mutations on the interactions between UL52, UL8, and UL5 in the absence of helicase-primase inhibitor. We found that the mutations S364G and R367H in UL52, alone or in combination with G352V and M351I in UL5, reduced the amount of GFP-UL52 in an HA-UL5 pulldown experiment (Fig. 5*a*). The mutations G352V and M351I in UL5 had no effects; mutations A899T in UL52 and K356T in UL5, on the other hand, moderately increased the amounts of eGFP-UL52 brought down by the anti-HA beads.

Effects of BAY 54-6322, which is structurally closely related to BAY 57-1293 (27), on the UL5-UL52 interaction were also examined in HA-UL5 co-immunoprecipitation experiments (Fig. 5, *b* and *c*). We observed a significant and dose-dependent increase in the efficiency by which eGFP-UL52 was co-immunoprecipitated with HA-UL5 in the presence of the drug (Fig. 5*d*). At very high concentrations of the drug, the total amount of soluble UL5, UL52, and UL8 in the extract decreased, but the efficiency of eGFP-UL52 co-immunoprecipitation still increased. In contrast, using the mutant versions, K356T in UL5 and A899T in UL52, we observed the opposite effect (Fig. 5*d*).

To verify the physiological effects of the drug and the drug resistance mutations, we examined transient HSV-1 oriS-dependent DNA replication in cells using expression plasmids and a plasmid containing HSV-1 oriS (Fig. 5*e*). We found that BAY 54-6322 inhibited DNA replication with an IC_50_ of 2 μm, whereas inhibition of replication in the experiment using mutant proteins required more than 10-fold higher concentrations of the drug.

##### Interactions between the Single Strand DNA-binding Protein ICP8, UL8, and the UL52 M-domain

**FIGURE 6.**
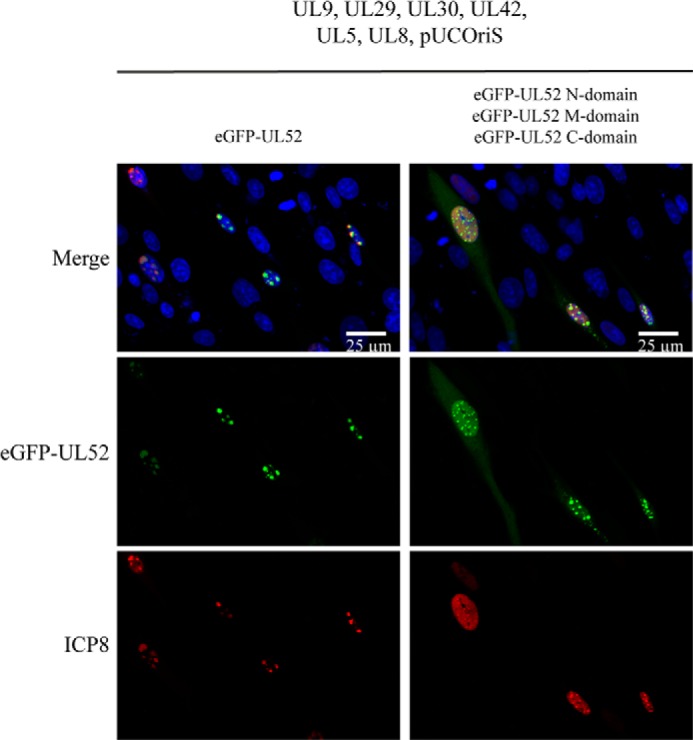
**Intranuclear foci formed by HSV-1 replication proteins in transfected cells.** Cultured cells were transfected with a plasmid containing HSV-1 oriS and either expression plasmids for eGFP-UL52 and the remaining six HSV-1 replication proteins (*left panels*) or expression plasmids encoding eGFP-tagged UL52 N-, M- and C-domains and the remaining six replication proteins (*right panels*). DNA was visualized with DAPI, ICP8 by immunostaining using the ab20194 antibody. Cells were subjected to confocal microscopy using a ×20/0.8 objective.

**FIGURE 7.**
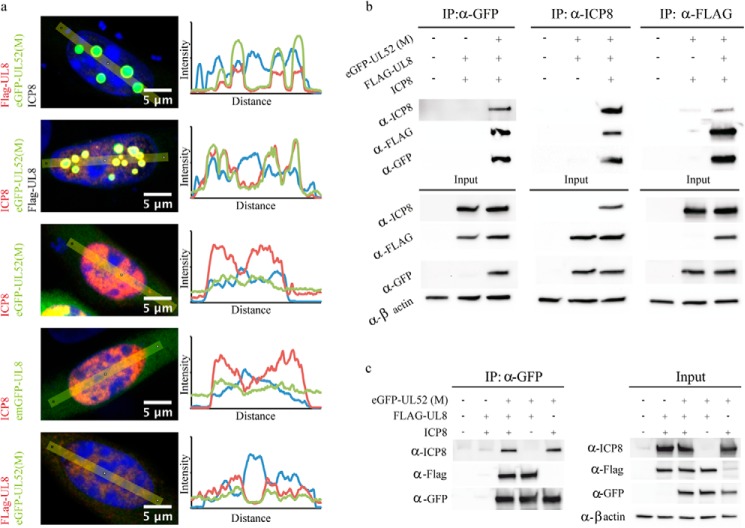
**Interactions between the UL52 M-domain, UL8, and ICP8.** *a,* co-localization in nuclei of transfected cells was visualized using the following: (i) anti-ICP8 antibodies (*red*) and eGFP UL52 M-domain (*green*); (ii) anti-FLAG antibodies (*red*) and eGFP-UL52 M-domain (*green*); and (iii) ICP8 antibodies (*red*) and emerald GFP (*emGFP*)-UL8 (*green*) as indicated. DNA was stained with DAPI (*blue*). Pictures were taken using confocal microscopy equipped with a ×63/1.4 oil objective. Profile plots were generated from measurements of the fluorescence signals with a linear scan through the nuclei. *b,* Western blot analysis of co-immunoprecipitation experiments performed in cell extracts containing transiently expressed FLAG-UL8, ICP8, and the eGFP-tagged UL52 M-domain as indicated. *c,* Western blot analysis of an anti-GFP co-immunoprecipitation experiment performed in cell extracts containing ICP8, eGFP-tagged UL52 M-domain and FLAG-UL8, as indicated.

As shown above, eGFP-UL52, HA-UL5, and FLAG-UL8 together with expression plasmids encoding UL9, UL29, UL30, and UL42 can fully support transient oriS-dependent DNA replication in transfected cells (Figs. 1*b* and 5*e*). We then examined whether the individual domains of UL52 all together could also support transient DNA replication, but we were unable to detect oriS-dependent DNA replication in this instance (data not shown). However, we observed, under these conditions, prominent intranuclear foci containing eGFP-tagged UL52 domains and ICP8, indicating co-localization of replication proteins (Fig. 6). We then performed a systematic search for the minimal requirement for formation of intranuclear foci by subtracting, one by one, each of the three domains and the remaining six replication proteins as well as the oriS-containing plasmid. The results unambiguously demonstrated that simultaneous expression of the eGFP-UL52 M-domain, FLAG-UL8, and ICP8 were sufficient to produce prominent intranuclear foci, and elimination of any of these components resulted in diffuse staining patterns (Fig. 7*a*). The results point toward the existence of complex(es) between the UL52 M-domain, UL8, and ICP8. We then performed co-immunoprecipitation experiments using anti-GFP, anti-ICP8, and anti-FLAG antibodies, respectively, in cell extracts containing eGFP-tagged UL52 M-domain, ICP8, and FLAG-tagged UL8 (Fig. 7*b*). Our results revealed that both FLAG-UL8 and ICP8 were co-immunoprecipitated with the eGFP-UL52 M-domain. Conversely, in a co-immunoprecipitate experiment using anti-ICP8, we noted that the UL52 M-domain as well as FLAG-UL8 were brought down together with ICP8; however, anti-FLAG antibodies pulled down FLAG-UL8 and the UL52 M-domain but very little ICP8 (Fig. 7*b*). Our results indicate that the UL52 M-domain can form separate complexes with UL8 and ICP8. To confirm the existence of a binary complex between the UL52 M-domain and ICP8, we performed co-immunoprecipitation experiments for ICP8 and the UL52 M-domain in the presence or absence of UL8 (Fig. 7*c*). Here, we found that anti-GFP antibodies co-immunoprecipitated the eGFP-tagged UL52 M-domain and ICP8 in the presence as well as absence of UL8. We thus conclude that the UL52 M-domain is capable of forming separate binary complexes with ICP8 and UL8.

To summarize, as shown above, a binary complex can be formed between UL8 and the UL52 M-domain (Fig. 2*a*). In addition, another binary complex between the UL52 M-domain and ICP8 also exists (Fig. 7, *b* and *c*). The formation of prominent intranuclear foci, which requires simultaneous expression of the UL52 M-domain, UL8 and ICP8, suggests that a ternary complex also might exist (Fig. 7*a*). The existence of a ternary complex is also supported by the observation that UL5-UL8-UL52 can bind ICP8 in a surface plasmon resonance experiment, whereas UL5-UL52 subcomplex cannot (20). One might speculate that one important role of UL8 is to provide access for UL52 to ICP8-coated single-stranded DNA by unveiling an ICP8-binding site on the UL52 M-domain. Biochemical experiments using purified proteins and DNA co-factors will ultimately be necessary to clarify the nature of these complexes and how they relate to primer synthesis on a lagging strand coated with ICP8.

## DISCUSSION

The herpesvirus helicase-primase complex is an essential component of the virus replisome, and it is conserved in all herpesviruses. The superfamily I DNA helicase UL5 is related to the cellular PifI helicase and interacts with UL52. UL52 is a member of the archaeo-eukaryotic primase superfamily with a zinc ribbon-like domain, and together with UL5, it forms a complex with helicase and primase activities (15, 16). An additional component, UL8, is required to form a fully functional complex that can support virus replication *in vivo*. UL8, which has been identified as an inactivated family B DNA polymerase (17), is required for interactions with the single-stranded DNA-binding protein ICP8. In the presence of UL8, helicase-primase can together with ICP8 support leading and lagging strand DNA synthesis *in vitro* and also form a complex with ICP8 as detected by surface plasmon resonance (20, 21). As part of a systematic effort to characterize the herpesvirus replisome, we have here investigated interactions between the UL52 primase and its partners UL8, UL5, and ICP8.

We have identified a UL52 N-domain, amino acids 1–421, which binds UL5. Amino acids between positions 367 and 421 in UL52 are essential for this interaction. The importance of this region is highlighted by the resistance mutations S364G and R367H in UL52 to the helicase-primase inhibitor ASP2151 (25), and we find that they destabilize the UL5-UL52 interaction. This region becomes sensitive to endoproteolytic cleavage in the presence of UL5, indicating the presence of a hinge region that can help to accommodate conformational strain resulting from movement of UL5 and UL52 in opposite directions on the lagging strand template.

The minimal M-domain of UL52, amino acids 422–887, contains the primase active site and binds to UL8. The M-domain is also involved in an interaction with ICP8 as demonstrated by the formation of prominent intranuclear foci containing the M-domain, UL8, and ICP8. Subtraction of any of these three components eliminates formation of nuclear foci suggesting a complex set of interactions between these three components. Here, we have identified separate binary complexes between the UL52 M-domain and UL8 and ICP8, respectively. The formation of prominent intranuclear foci containing the three components may indicate the formation of insoluble ICP8 filaments capable of attracting the M-domain and its partner UL8 (28). A direct interaction between ICP8 and UL8 remains to be identified. Also, a ternary complex formed by soluble UL52 M-domain, UL8, and ICP8 may still exist but remains to be identified.

The functional properties of these complexes may reveal how UL52 gets access to ICP8-coated single-stranded DNA for primer synthesis. Clearly, biochemical characterization of these complexes together with crystallography will be required for a more complete understanding of priming promoted by the helicase-primase complex.

The role of the C-domain is less clear. Mutations in the zinc-binding motif render the UL5-UL52 subcomplex enzymatically inactive with no helicase and primase activities as well as severely reduced single strand DNA ATPase activity (29). Further studies have indicated that the zinc-binding domain is involved in DNA binding by UL52 and possibly loading of UL5 (30). These observations indicate the existence of direct interactions between the UL52 C-domain and UL5. The fact that resistance to structurally highly related drugs such as BAY 54-6322 and BAY 57-1293 may involve mutations both in UL5, K356T, and UL52, A899T, prompted us to look at effects of the drug and the drug resistance mutations on complex formation (26). BAY 54-6322 greatly stabilizes the UL5-UL52 complex, and resistance mutations completely obliterate this effect. Possibly, the drug will trap UL5 in a conformation favoring an interaction with UL52, and resistance may be explained by decreased affinity of UL5 for the drug. However, because the UL52 A899T mutation in combination with the UL5 K356T mutation further enhances drug resistance 25-fold *in vivo* as compared with the UL5 mutation alone, it is possible that the interaction between the two proteins also involves the C-domain (26). This interpretation is further supported by our observation that expression of the C-domain appears to increase UL5 solubility (Fig. 3*a*). It is therefore a possibility that a second UL5-binding site may involve neighboring structures from the UL52 M- and C-domains.

Taken together, our results facilitate future investigations of the structure and function of helicase-primase subcomplexes and thereby provide new insights into the mechanisms by which helicase-primase inhibitors interfere with herpesvirus replication. It is to be expected that such information should also assist continued development of antiviral compounds.

Finally, the herpesvirus replisome is highly conserved in evolution, but it can be assembled by different mechanism as shown by the different initiation mechanisms as observed for alpha-, beta-, and gammaherpesviruses. As recently suggested, the helicase-primase is derived from cellular ancestors to Pif1 helicase, Primpol, and a family B DNA polymerase, and it may have retained some of the functional properties once performed in cells (17). Further structural and functional studies of helicase-primase may therefore help to explain the evolution of herpesviruses.

## Footnotes

2 The abbreviations used are:

BHK: baby hamster kidney
eGFP: enhanced green fluorescent protein.

## References

[1] Elion G. B. (1983) The biochemistry and mechanism of action of acyclovir. J. Antimicrob. Chemother. 12, Suppl. B, 9–17 6313600 10.1093/jac/12.suppl_b.9

[2] Elion G. B. (1993) Acyclovir: discovery, mechanism of action, and selectivity. J. Med. Virol. Suppl. 1, 2–6 8245887 10.1002/jmv.1890410503

[3] Reardon J. E. Spector T. (1989) Herpes simplex virus type 1 DNA polymerase: mechanism of inhibition by acyclovir triphosphate. J. Biol. Chem. 264, 7405–7411 2540193

[4] Hanes J. W. Zhu Y. Parris D. S. Johnson K. A. (2007) Enzymatic therapeutic index of acyclovir. Viral *versus* human polymerase gamma specificity. J. Biol. Chem. 282, 25159–25167 17573351 10.1074/jbc.M703972200

[5] Danve-Szatanek C. Aymard M. Thouvenot D. Morfin F. Agius G. Bertin I. Billaudel S. Chanzy B. Coste-Burel M. Finkielsztejn L. Fleury H. Hadou T. Henquell C. Lafeuille H. Lafon M. E. Le Faou A. Legrand M. C. Maille L. Mengelle C. Morand P. Morinet F. Nicand E. Omar S. Picard B. Pozzetto B. Puel J. Raoult D. Scieux C. Segondy M. Seigneurin J. M. Teyssou R. Zandotti C. (2004) Surveillance network for herpes simplex virus resistance to antiviral drugs: 3 year follow-up. J. Clin. Microbiol. 42, 242–249 14715760 10.1128/JCM.42.1.242-249.2004 PMC321677

[6] Stránská R. Schuurman R. Nienhuis E. Goedegebuure I. W. Polman M. Weel J. F. Wertheim-Van Dillen P. M. Berkhout R. J. van Loon A. M. (2005) Survey of acyclovir-resistant herpes simplex virus in the Netherlands: prevalence and characterization. J. Clin. Virol. 32, 7–18 15572000 10.1016/j.jcv.2004.04.002

[7] Weller S. K. Kuchta R. D. (2013) The DNA helicase-primase complex as a target for herpes viral infection. Expert Opin. Ther. Targets 17, 1119–1132 23930666 10.1517/14728222.2013.827663 PMC4098783

[8] Wald A. Corey L. Timmler B. Magaret A. Warren T. Tyring S. Johnston C. Kriesel J. Fife K. Galitz L. Stoelben S. Huang M. L. Selke S. Stobernack H. P. Ruebsamen-Schaeff H. Birkmann A. (2014) Helicase-primase inhibitor pritelivir for HSV-2 infection. N. Engl. J. Med. 370, 201–210 24428466 10.1056/NEJMoa1301150

[9] Wu C. A. Nelson N. J. McGeoch D. J. Challberg M. D. (1988) Herpes simplex virus type 1 gene products required for DNA replication: identification and overexpression. J. Virol. 62, 435–443 2535726 10.1128/jvi.63.1.196-204.1989 PMC247673

[10] Muylaert I. Tang K-W. Elias P (2011) Replication and recombination of herpes simplex virus DNA. J. Biol. Chem. 286, 15619–15624 21362621 10.1074/jbc.R111.233981 PMC3091170

[11] Aslani A. Olsson M. Elias P. (2002) ATP-dependent unwinding of a minimal origin of DNA replication by the origin binding protein and the single strand DNA-binding protein ICP8 from herpes simplex virus type1. J. Biol. Chem. 277, 41204–41212 12183471 10.1074/jbc.M208270200

[12] Muylaert I. Zhao Z. Andersson T. Elias P. (2012) Identification of conserved amino acids in the herpes simplex virus type 1 UL8 protein required for DNA synthesis and UL52 primase interaction in the virus replisome. J. Biol. Chem. 287, 33142–33152 22851167 10.1074/jbc.M112.356782 PMC3460421

[13] Crute J. J. Mocarski E. S. Lehman I. R. (1988) A DNA helicase induced by herpes simplex virus type 1. Nucleic Acids Res. 16, 6586–6596 10.1093/nar/16.14.6585 PMC338315 2840645

[14] Crute J. J. Tsurumi T. Zhu L. A. Weller S. K. Olivo P. D. Challberg M. D. Mocarski E. S. Lehman I. R. (1989) Herpes simplex virus 1 helicase-primase: a complex of three herpes-encoded gene products. Proc. Natl. Acad. Sci. U.S.A. 86, 2186–2689 2538835 10.1073/pnas.86.7.2186 PMC286876

[15] Hodgman T. C. (1988) A new superfamily of replicative proteins. Nature 333, 22–23 3362205 10.1038/333022b0

[16] Iyer L. M. Koonin E. V. Leipe D. D. Aravind L. (2005) Origin and evolution of the archaeo-eukaryotic primase superfamily and related palm-domain proteins: structural insights and new members. Nucleic Acids Res. 33, 3875–3896 16027112 10.1093/nar/gki702 PMC1176014

[17] Kazlauskas D. Venclovas C. (2014) Herpesviral helicase-primase subunit UL8 is inactivated B-family polymerase. Bioinformatics 30, 2093–2097 24747220 10.1093/bioinformatics/btu204

[18] Calder J. M. Stow N. D. (1990) Herpes simplex virus helicase-primase: the UL8 protein is not required for DNA-dependent ATPase and DNA helicase activities. Nucleic Acids Res. 18, 3573–3578 2163521 10.1093/nar/18.12.3573 PMC331012

[19] Dodson M. S. Lehman I. R. (1991) Association of DNA helicase and primase activities with a subassembly of the herpes simplex virus type 1 helicase-primase composed of the UL5 and UL52 gene products. Proc. Natl. Acad. Sci. U.S.A. 88, 1105–1109 1847509 10.1073/pnas.88.4.1105 PMC50965

[20] Falkenberg M. Bushnell D. A. Elias P. Lehman I. R. (1997) The UL8 subunit of the heterotrimeric herpes simplex virus type 1 helicase-primase is required for the unwinding of single strand DNA-binding protein (ICP8)-coated DNA substrates. J. Biol. Chem. 272, 22766–22770 9278436 10.1074/jbc.272.36.22766

[21] Falkenberg M. Lehman I. R. Elias P. (2000) Leading and lagging strand DNA synthesis *in vitro* by a reconstituted herpes simplex virus type 1 replisome. Proc. Natl. Acad. Sci. U.S.A. 97, 3896–3900 10760262 10.1073/pnas.97.8.3896 PMC18113

[22] Cavanaugh N. A. Ramirez-Aguilar K. A. Urban M. Kuchta R. D. (2009) Herpes simplex virus-1 helicase-primase: roles of each subunit in DNA binding and phosphodiester bond formation. Biochemistry 48, 10199–10207 19788334 10.1021/bi9010144 PMC2783883

[23] Constantin N. Dodson M. S. (1999) Two-hybrid analysis of the interaction between the UL52 and UL8 subunits of the herpes simplex virus type 1 helicase-primase. J. Gen. Virol. 80, 2411–2415 10501495 10.1099/0022-1317-80-9-2411

[24] Stow N. D. Hammarsten O. Arbuckle M. I. Elias P. (1993) Inhibition of herpes simplex virus type 1 DNA replication by mutant forms of the origin-binding protein. Virology 196, 413–418 8396795 10.1006/viro.1993.1496

[25] Chono K. Katsumata K. Kontani T. Shiraki K. Suzuki H. (2012) Characterization of virus strains resistant to the herpes virus helicase-primase inhibitor ASP2151 (Amenamevir). Biochem. Pharmacol. 84, 459–467 22687623 10.1016/j.bcp.2012.05.020

[26] Biswas S. Kleymann G. Swift M. Tiley L. S. Lyall J. Aguirre-Hernández J. Field H. J. (2008) A single drug-resistance mutation in HSV-1 UL52 primase points to a difference between two helicase-primase inhibitors in their mode of interaction with the antiviral target. J. Antimicrob. Chemother. 61, 1044–1047 18299638 10.1093/jac/dkn057

[27] Kleymann G. Fischer R. Betz U. A. Hendrix M. Bender W. Schneider U. Handke G. Eckenberg P. Hewlett G. Pevzner V. Baumeister J. Weber O. Henninger K. Keldenich J. Jensen A. Kolb J. Bach U. Popp A. Mäben J. Frappa I. Haebich D. Lockhoff O. Rübsamen-Waigmann H. (2002) New helicase-primase inhibitors as drug candidates for the treatment of herpes simplex disease. Nat. Med. 8, 392–398 11927946 10.1038/nm0402-392

[28] O'Donnell M. E. Elias P. Funnell B. E. Lehman I. R. (1987) Interaction between the DNA polymerase and single strand DNA-binding protein (infected cell protein 8) of herpes simplex virus. J. Biol. Chem. 262, 4260–4266 3031068

[29] Biswas N. Weller S. K. (1999) A mutation in the C-terminal putative Zn^2+^ finger motif of UL52 severely affects the biochemical activities of the HSV-1 helicase-primase subcomplex. J. Biol. Chem. 274, 8068–8076 10075707 10.1074/jbc.274.12.8068

[30] Chen Y. Carrington-Lawrence S. D. Bai P. Weller S. K. (2005) Mutations in the putative zinc-binding motif of UL52 demonstrates a complex interdependence between UL5 and UL52 subunits of the human herpes simplex virus type 1 helicase/primase complex. J. Virol. 79, 9088–9096 15994803 10.1128/JVI.79.14.9088-9096.2005 PMC1168741

[31] Zhu L. A. Weller S. K. (1992) The six conserved helicase motifs of the UL5 gene product, a component of the herpes simplex virus type 1 helicase-primase, are essential for function. J. Virol. 66, 469–479 1309257 10.1128/jvi.66.1.469-479.1992 PMC238307

